# Endo-Perio Lesion and Uncontrolled Diabetes

**DOI:** 10.1155/2018/7478236

**Published:** 2018-05-16

**Authors:** Sara Dhoum, Kaoutar Laslami, Fatimazahraa Rouggani, Amal El Ouazzani, Mouna Jabri

**Affiliations:** Department of Conservative Dentistry and Endodontics, School of Dentistry of Casablanca, Casablanca, Morocco

## Abstract

This work is to discuss the management of an endo-perio lesion, which represents a challenge to clinicians when it comes to diagnosis and prognosis of the involved teeth and especially with an altered general condition. A 50-year-old female patient with uncontrolled diabetes type 2 is suffering from a purulent discharge coming from the upper right canine. Endodontic and periodontal treatments were realized with 36 months radiological and clinical follow-up with the collaboration of her internist doctor.

## 1. Introduction

The term “endo-perio” lesion emerged decades ago to designate a specific disease condition affecting the pulp and the periodontal tissues simultaneously [1, 2].

Diabetic patients are more exposed to oral infections and periradicular lesions caused by the changes of their immune system, qualitative and quantitative changes in normal flora of their oral cavity, and poor peripheral blood supply.

The pulp properties are changing with the aging process; moreover, uncontrolled diabetes can cause changes of the dental pulp tissue and reduce its activity by reducing the collateral blood flow. Since diabetes damages the blood circulation or causes ischemia, sometimes necrosis of the pulp may occur [3, 4].

The possible connection between chronical oral inflammatory processes, such as apical periodontitis, endodontic state, and systemic health, is one of the most interesting aspects faced by medical and dental scientific community, by monitoring the potential of healing after stabilization of all the parameters, in the present case, the inflammation and the infection states in a diabetic ground.

## 2. Case Report

**Figure 1:**
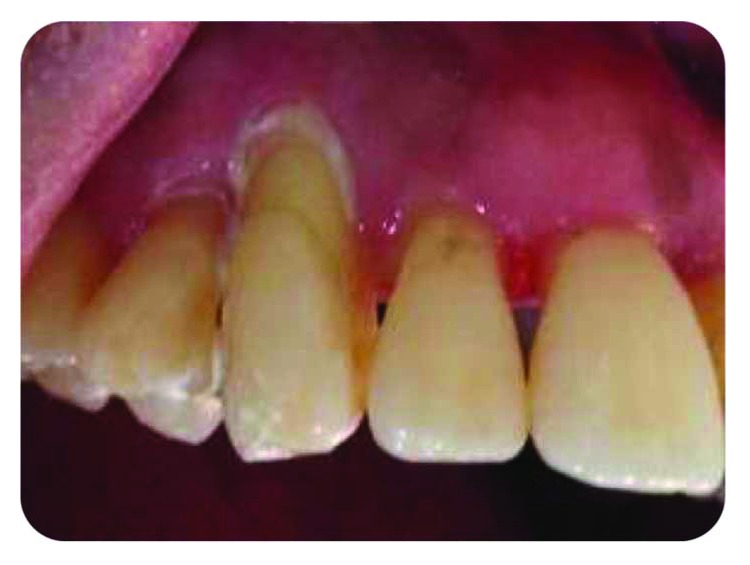
Purulent discharge related to number 13.

A 50-year-old female patient, with uncontrolled type 2 diabetes, is suffering from a purulent discharge coming from number 13 sulcus, with dental mobility (grade 3) and no apparent decay, fracture, or fissure (Figure 1).

**Figure 2:**
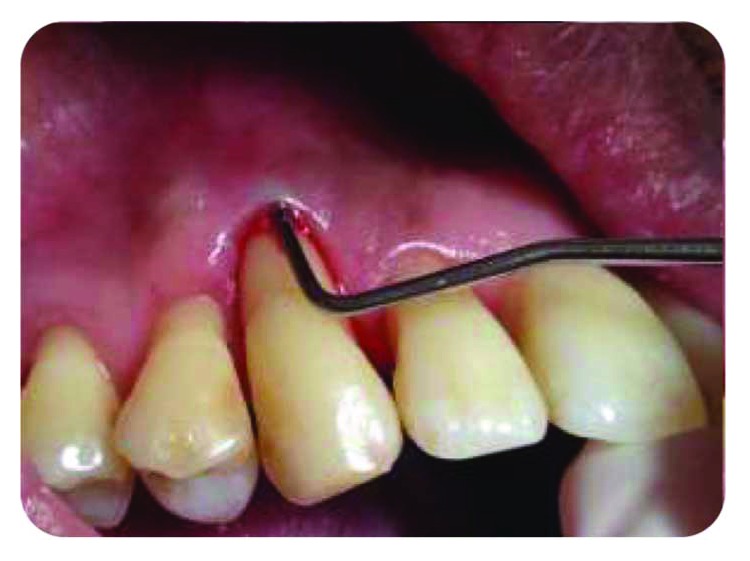
Punctual periodontal probing, 12 mm periodontal pocket.

*Initial periodontal treatment*: nonresponsive (Figure 2).

*The sensitivity tests*: all negative.

**Figure 3:**
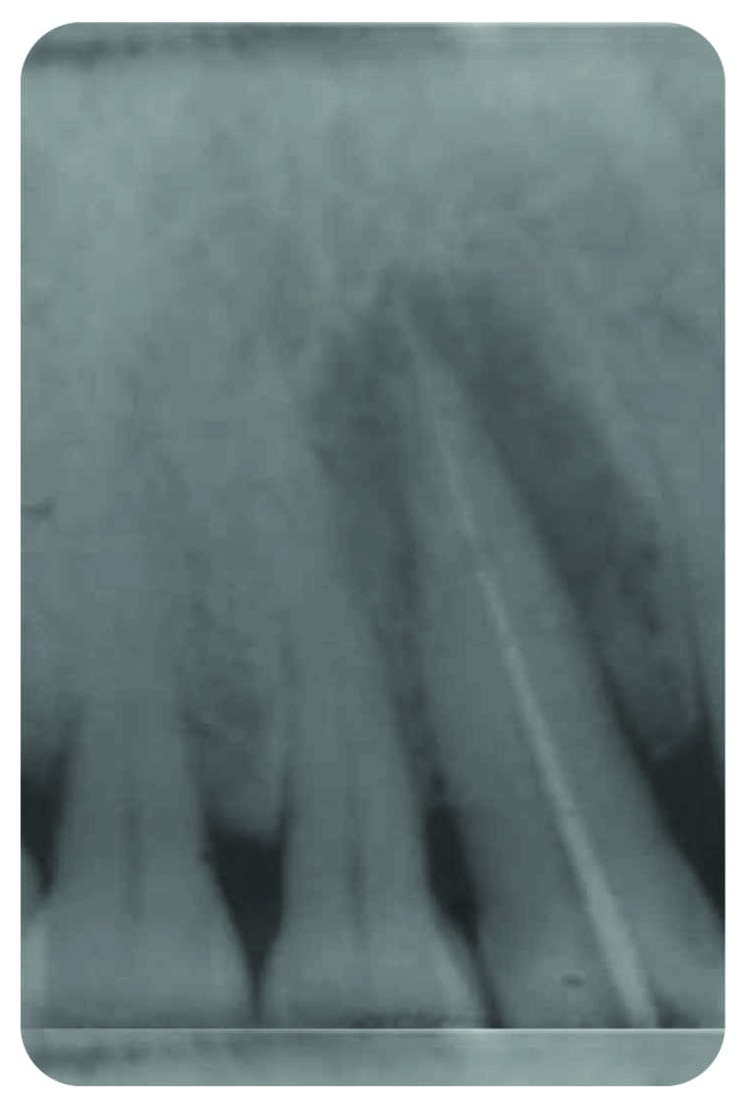
Initial state of the periodontal area.

*Radiography*: severe bone loss related to a periapical lesion (Figure 3).

## 3. Diagnosis

Diagnoses of the patient were primary periodontal disease with secondary endodontic involvement and chronic generalized periodontitis as a manifestation of systemic diseases (diabetes) (American Academy of Periodontology Classification, 1999).

### 3.1. Therapeutic Decision

Therapeutic decision was made after management of periodontal disease with scaling and root planning, patient education, and a program of periodontal hygiene maintenance (the protocol followed is the one recommended by Abbott [1]).

**Figure 4:**
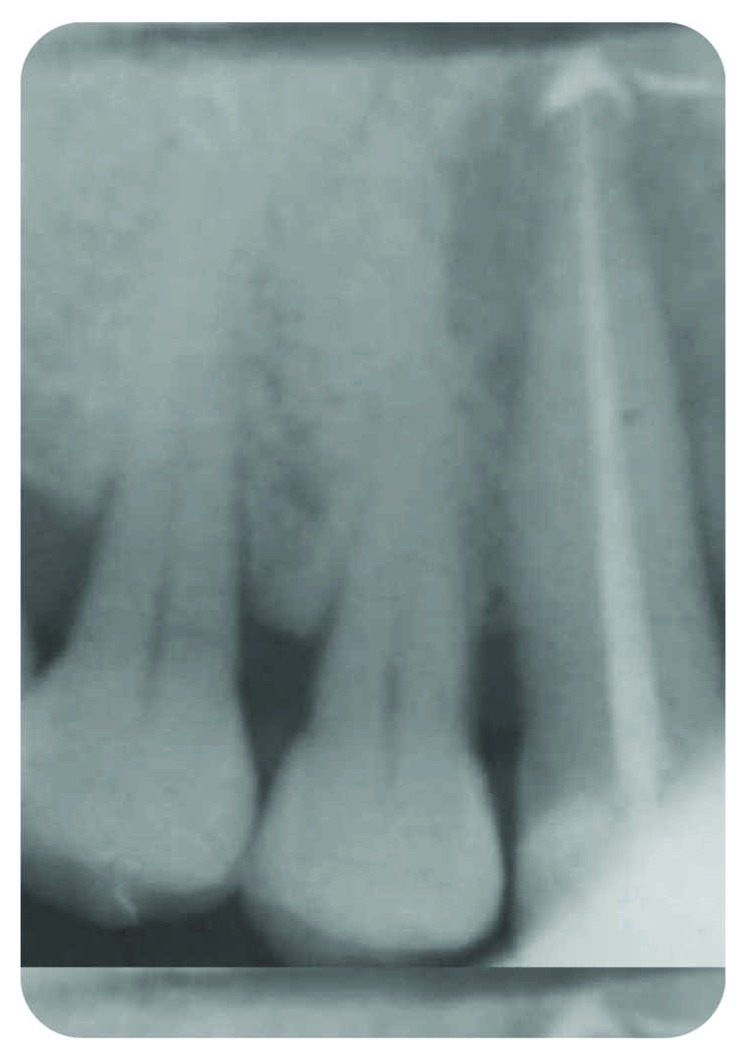
RCT and tridimensional filling with Thermafil—suspicion of lateral canal.

Endodontic treatment was administrated in two visits:

1. First appointment
  - Patient under amoxicillin medication two days before the RCT treatment and the week following the procedure (collaboration with her internist doctor)
  - Realization of the access cavity under a dental dam and without local anesthesia
  - Mechanical preparation of the root canal system using ProTaper Universal® rotary system (Dentsply International)
  - Chemical disinfection using 2,5% sodium hypochlorite
  - Temporary filling of the root canal with calcium hydroxide
  - Placement of an adequate temporary coronal filling (Cavit™ 3M ESPE)
2. Second appointment
  - Adequate mechanical debridement of the root canal using stainless steel K-files combined to ProTaper Universal rotary files
  - Irrigation using 2,5% sodium hypochlorite
  - Proper drying of the root canal using sterile paper cones
  - Tridimensional root canal obturation using Thermafil® (Dentsply Maillefer) (Figure 4)

During the preparation of the root canal system since the first appointment, there was no exudation coming from the root canal.

### 3.2. Two-Week Follow-Up (Figure 5)

- Beginning of bone reorganization
- Decrease but without disappearance of the purulent discharge
- Decrease of dental mobility
- Stabilization of the patient's blood sugar level with the collaboration of the internist doctor

### 3.3. Two-Month Follow-Up (Figure 6)

**Figure 7:**
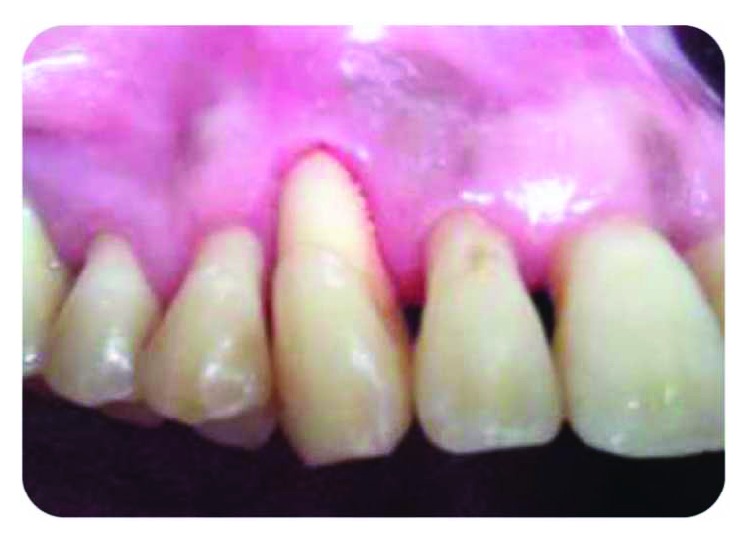
Beginning of soft tissue healing.

- Stabilization of the radiolucent image revealed by radiographic examination
- Beginning of bone reorganization
- Decrease but without disappearance of the purulent discharge (Figure 7)

### 3.4. Six-Month Follow-Up

**Figure 8:**
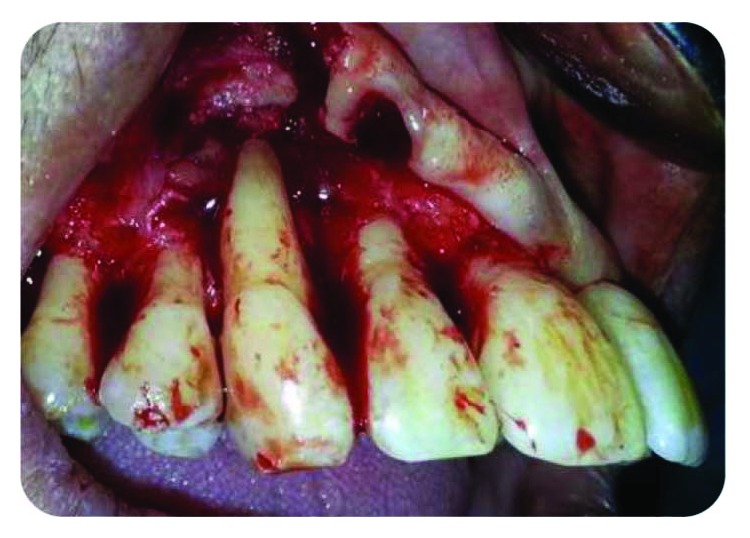
Open flap of upper right quadrant.

**Figure 9:**
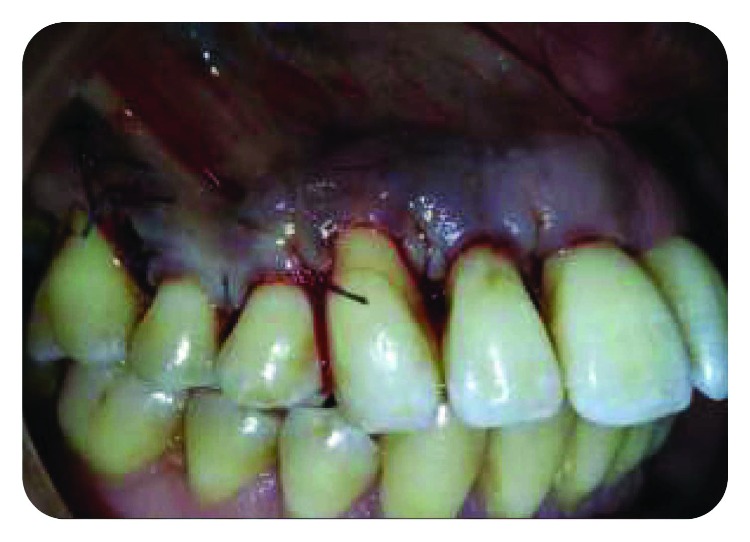
Operative site after suturing.

**Figure 10:**
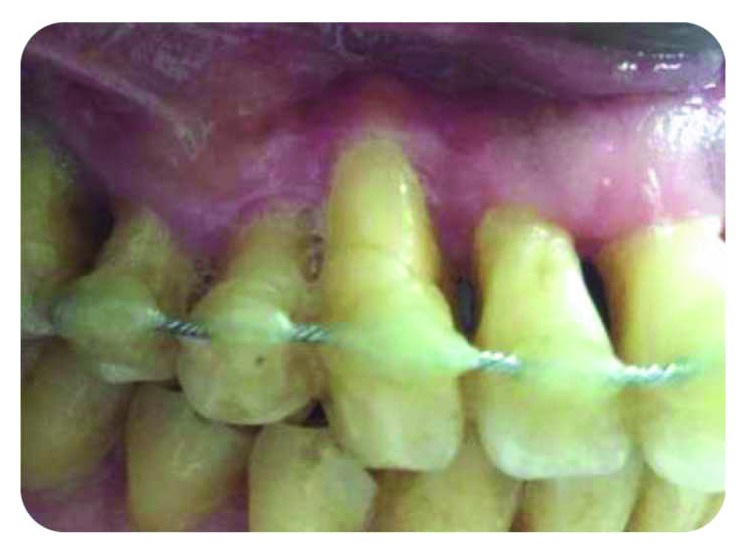
Soft tissue healing and periodontal splinting.

- Persistence of the purulent discharge from the sulcus was noted.
- An open flap for periodontal cleaning was realized with a debridement of the root surface, and a full periodontal therapy was established to complete the treatment and to obtain a periodontal attachment repair (Figures 8 and 9).
- Periodontal splinting was used to reinforce and improve the healing potential of the tooth in question (Figure 10).

#### 3.4.1. The Six Months' Recall Revealed

**Figure 11:**
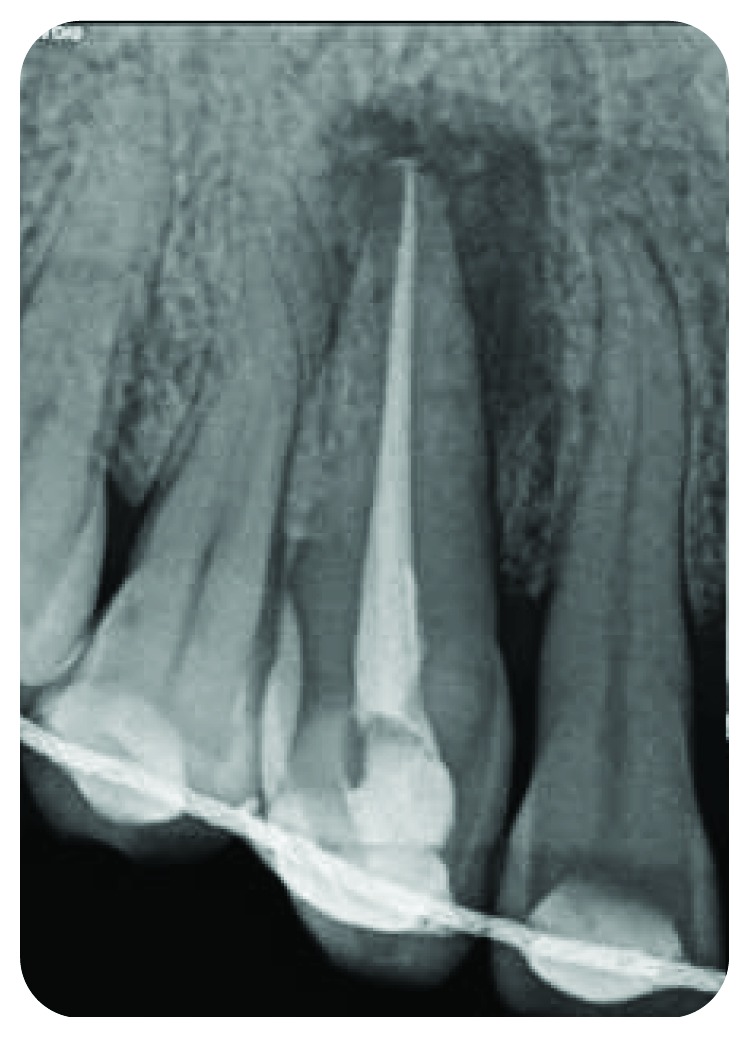
Six months' recall radiograph.

- Soft tissue healing with gingival recession located on number 13 (Figure 10);
- A complete disappearance of the purulent production;
- Partial bone regeneration with apparent bone trabeculations in the former radiolucency (Figure 11).

#### 3.4.2. At 18 Months and 39 Months' Recall

**Figure 12:**
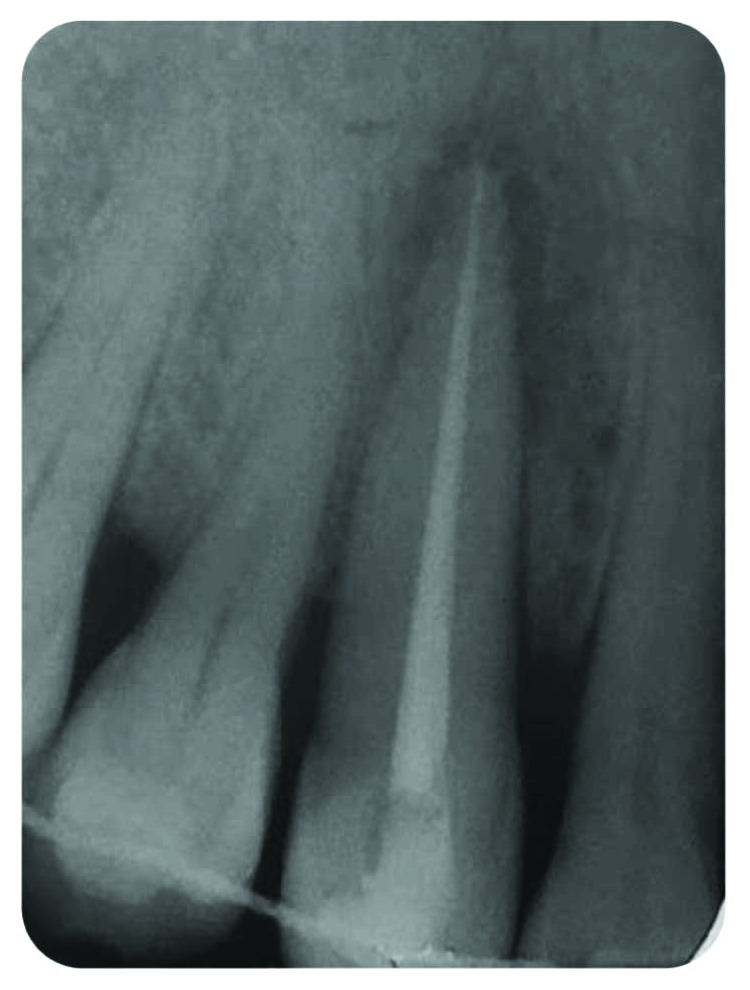
18 months' recall radiograph.

**Figure 13:**
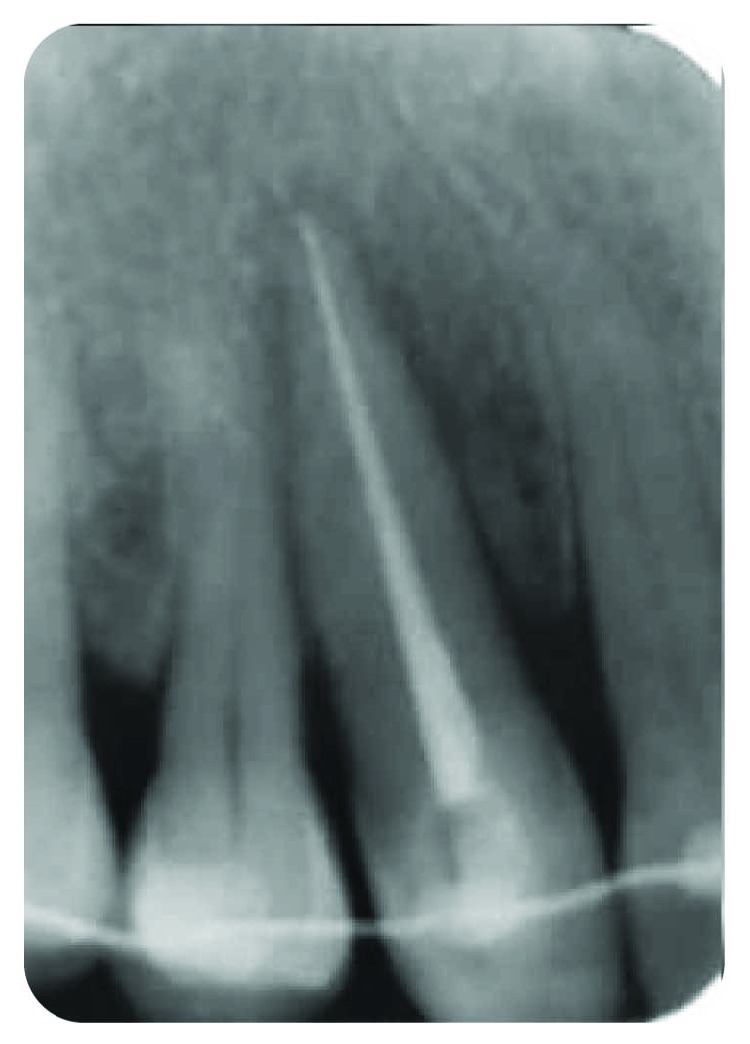
39 months' recall radiograph showing a bone reorganization.

**Figure 14:**
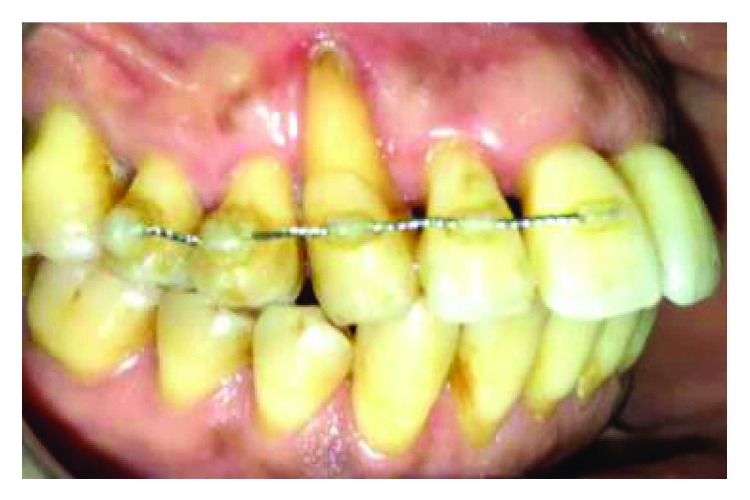
39 months' recall check-up showing a complete disappearance of the purulent discharge with a buccal bone loss and gingival recession regarding number 13.

- A progressive bone healing after 18 months was observed, then 39 months with a disappearance of the former radiolucency (Figures 12, 13, and 14).

**Figure 15:**
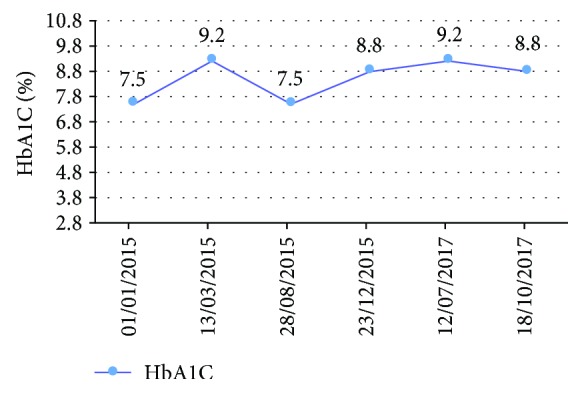
HbA1C follow-up from 2015 to October 2017.

This glycosylated hemoglobin curve demonstrates that the patient has an imbalanced diabetes (the treatment was established on February 2015.) (Figure 15).

## 4. Discussion

Diagnosis of primary endodontic disease and primary periodontal disease usually represents no clinical difficulty [2, 5]. Many classifications of endo-perio lesion are found in the literature; Simon et al. (1972) used a classification to separate lesions involving both periodontal and pulpal tissues into the following groups:

- Primary endodontic lesions with secondary periodontal involvement
- Primary periodontal lesions with secondary endodontic involvement
- True combined lesions

Many researches have reported substantial pathological change and frequent necrosis in the pulp tissue due to periodontal disease, especially with the presence of accessory canals [3, 6]. Other studies have stated that pulps in periodontally affected teeth remain within normal limits regardless of the severity of the periodontal pathosis and suggested that the systemic condition of the patient may have a big influence on the condition of the pulp than the status of the periodontal tissue or his chronologic age [4, 7].

Langeland et al. [8] have demonstrated that pathologic changes do occur in the pulp when periodontal disease is present; however, the pulp does not succumb as long as the apical foramen is not involved. It therefore seems evident that periodontal disease rarely jeopardizes the vital functions of the pulp unless the disease process has reached a terminal stage and involves the main pulpal blood supply, the apical area [9–11].

In diabetic patients, aging changes of pulp due to limited collateral blood flow are faster than nondiabetics. Since diabetes damages the blood circulation or ischemia, sometimes necrosis of pulp may occur [5, 12].

The prognosis depends on the differential diagnosis, the general state of the patient, the endodontic involvement, and the lesion age.

Diabetes mellitus (DM) is a significant and increasing global health problem. In 2013, the International Diabetes Federation estimated that there were 382 million people worldwide with diabetes increasing to 592 million in 2015 with the major part of this population is living in low- and middle-income countries [13, 14].

It is defined as a group of metabolic disorders characterized by chronic hyperglycaemia with disturbances in carbohydrates, fat, and protein metabolism resulting from defects in insulin secretion, insulin action on the target tissues, or both, and it is frequently associated with an increased susceptibility to infection [15–17].

Both diabetes mellitus type 1 (DM1) and type 2 (DM2) present numerous possible long-term complications. Epidemiological studies indicate that the severity of diabetic complications is generally proportional to the degree and duration of the hyperglycaemia.

Among the oral manifestations related to DM described are dry mouth, tooth decay, periodontal disease and gingivitis, oral candidiasis, burning mouth syndrome (BMS), taste disorders, rhinocerebral zygomycosis (mucormycosis), aspergillosis, oral lichen planus, geographic tongue and fissured tongue, delayed wound healing and increased incidence of infection, salivary dysfunction, altered taste and other neurosensory disorders, impaired tooth eruption, and benign parotid hypertrophy. Similar to the periodontium, the dentin-pulp complex is also affected by diabetes. Zehnder et al. [10] reported that angiopathy represented by a thickened basement membrane was observed in the dental pulp of diabetics. But still no systematic studies about the direct effect of the diabetes on the pulp tissue.

Periodontal healing after a proper RCT depends on the biological constants including the rate of blood sugar levels. Clinical and radiological follow-up must be completed with glycemic control which reveals in our case a chronic uncontrolled diabetes with regular visit controls with the internist doctor [18–20].

Lesion's age makes the prognosis of an endo-perio lesion much more uncertain, in periodontal pocket, complicated the lesion management. Bacterial ecosystem of chronic lesion adapts itself and becomes more resistant to endodontic and periodontal treatments [19, 21, 22].

Healing potential of an endodontic lesion is very high if the lesion is surrounded with 5 or 6 bone walls (Machtou and Cohen, 1988). According to Ng et al., Kambale et al., and Rudranaik et al. “the larger is the part caused by pulpal infection, the better the prognosis of attachment regeneration” [23–25].

## 5. Conclusion

The primary goal of all treatment must be to rid the patient of the infection.

Endodontic treatment success criteria were established in 1994 by the European Society of Endodontology and included the following:

- Absence of pain, swelling, and fistula
- Maintenance of tooth function
- Presence of radiological evidence of a normal periodontal ligament space
- Absence of apical periodontitis or radicular resorption

It can be stated that, in the majority of the endo-perio lesions, the bacterial etiology dictates the clinical course of the disease and therefore the treatment plan.

Changes in the immune system defense in diabetic patients against infection and pathological changes in pulp and periradicular tissue, lack of awareness of patients about the effect of diabetes on oral health, asymptomatic dental infections, poor dental and oral health, and lack of regular visit to dentists because of its high costs all may have influence.

Other factors, such as patient cooperation, restorability, and economics, will influence treatment decisions.

Neither periodontic nor endodontic treatment can be considered in isolation; clinically, they are closely related, and this must influence the diagnosis and treatment.

The clinical burden of diabetes is high in Morocco, and the majority of patients do not achieve the recommended glycaemia target, suggesting that there is a huge gap between evidence-based diabetic management and real-life practice. Better education of patients and improved compliance with international recommendations are necessary to deliver a better quality of diabetic care [14].

## Figures and Tables

**Figure 5:**
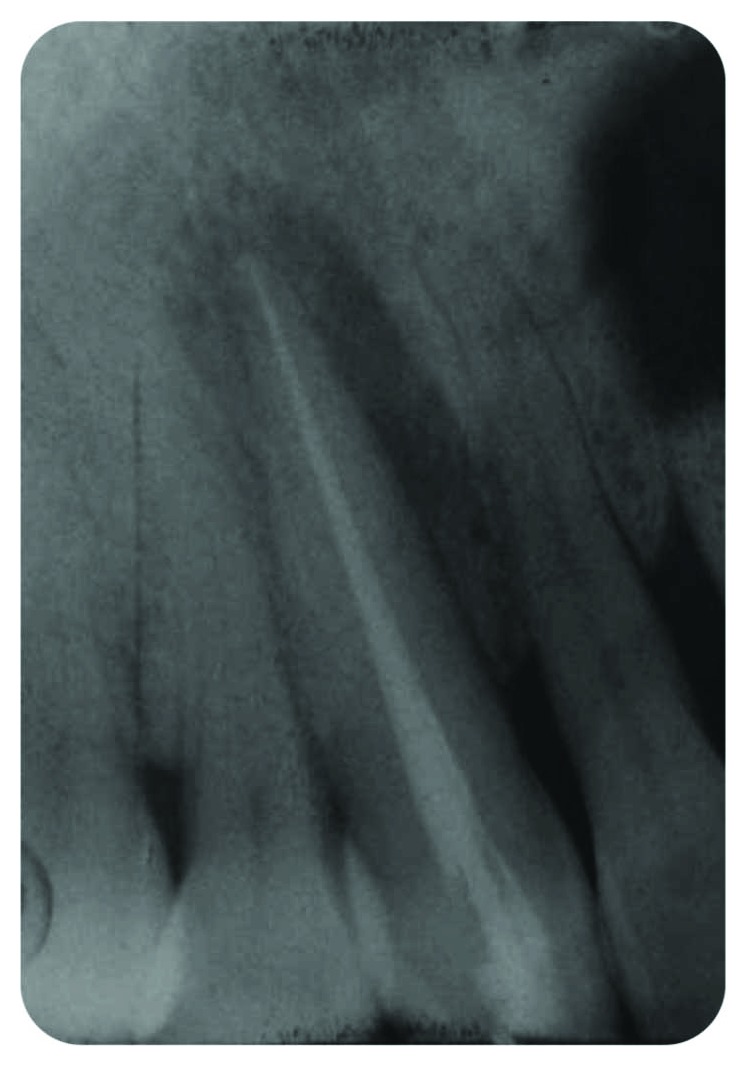
Disappearance of sealer puff two weeks after the endodontic therapy.

**Figure 6:**
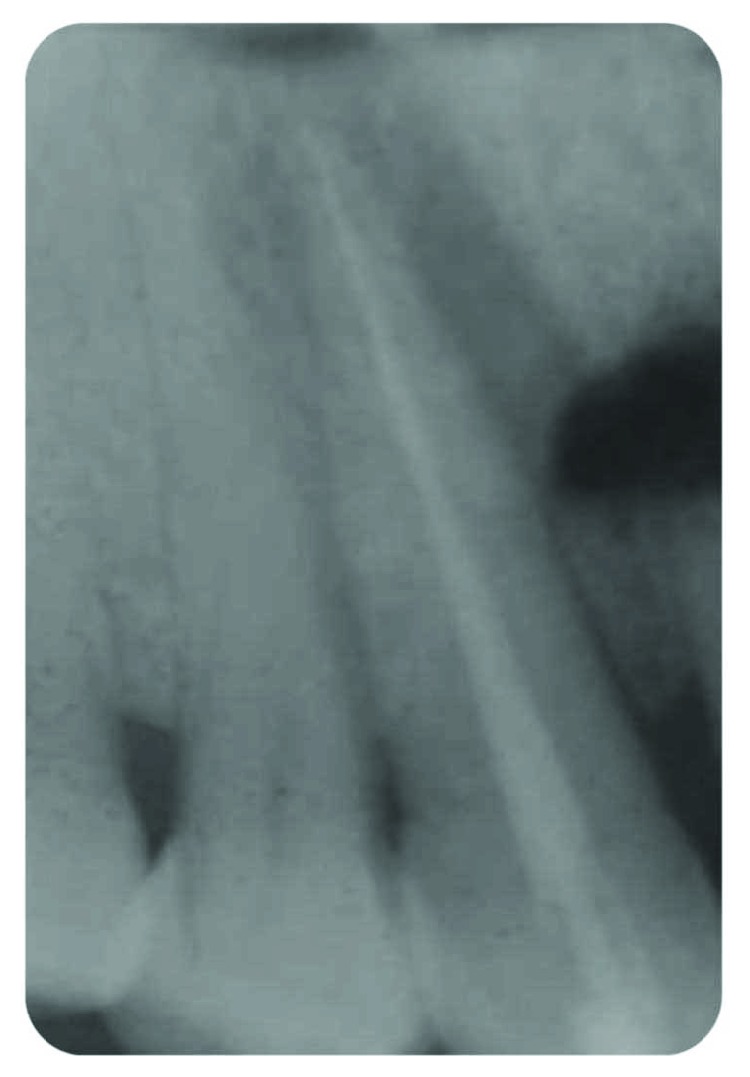
Two months' recall radiograph showing a continuous bone reorganization.
